# Differential neuroglycan C expression during retinal degeneration in *Rpe65*^−/−^ mice

**Published:** 2008-11-26

**Authors:** Pascal Escher, Sandra Cottet, Saichiko Aono, Atsuhiko Oohira, Daniel F. Schorderet

**Affiliations:** 1 Institute for Research in Ophthalmology, Sion, Switzerland; 2 Department of Ophthalmology, University of Lausanne, Lausanne, Switzerland; 3 Department of Perinatology, Institute for Developmental Research, Aichi Human Service Center, Kasugai, Japan; 4 EPFL-Ecole Polytechnique Fédérale, Lausanne, Switzerland

## Abstract

**Purpose:**

An increased mRNA expression of the genes coding for the extracellular matrix proteins neuroglycan C (*NGC*), interphotoreceptor matrix proteoglycan 2 (*IMPG2*), and CD44 antigen (*CD44*) has been observed during retinal degeneration in mice with a targeted disruption of the *Rpe65* gene (*Rpe65*^−/−^ mouse). To validate these data, we analyzed this differential expression in more detail by characterizing retinal *NGC* mRNA isoform and protein expression during disease progression.

**Methods:**

Retinas from C57/Bl6 wild-type and *Rpe65*^−/−^ mice, ranging 2 to 18 months of age, were used. *NGC*, *IMPG2*, and *CD44* mRNA expression was assessed by oligonucleotide microarray, quantitative PCR, and in situ hybridization. Retinal NGC protein expression was analyzed by western blot and immunohistochemistry.

**Results:**

As measured by quantitative PCR, mRNA expression of *NGC* and *CD44* was induced by about 2 fold to 3 fold at all time points in *Rpe65*^−/−^ retinas, whereas initially 4 fold elevated *IMPG2* mRNA levels progressively declined. *NGC* and *IMPG2* mRNAs were expressed in the ganglion cell layer, the inner nuclear layer, and at the outer limiting membrane. *NGC* mRNA was also detected in retinal pigment epithelium cells (RPE), where its mRNA expression was not induced during retinal degeneration. *NGC-I* was the major isoform detected in the retina and the RPE, whereas *NGC-III* was barely detected and *NGC-II* could not be assessed. NGC protein expression was at its highest levels on the apical membrane of the RPE. NGC protein levels were induced in retinas from 2- and 4-month-old *Rpe65*^−/−^ mice, and an increased amount of the activity-cleaved NGC ectodomain containing an epidermal growth factor (EGF)-like domain was detected.

**Conclusions:**

During retinal degeneration in *Rpe65*^−/−^ mice, NGC expression is induced in the neural retina, but not in the RPE, where NGC is expressed at highest levels.

## Introduction

Leber congenital amaurosis (LCA) is a genetically heterogeneous retinal dystrophy with prenatal onset. A subset of patients carries mutations in the retinal pigment epithelium protein of 65 kDa (*RPE65*) gene (LCA2; OMIM #204100) [1,2]. RPE65  is the iron (II)-dependent isomerohydrolase essential for the generation of the photopigment 11-cis retinal from all-trans-retinyl ester in the retinoid visual cycle [3-6]. In the RPE of mice with a targeted disruption of the *Rpe65* gene (*Rpe65*^−/−^ mice), no 11-*cis* retinal has been synthesized, and excessive accumulation of all-*trans* retinyl esters has been observed [7]. This enzymatic defect in the RPE was found to result in profound effects in the underlying photoreceptors. Cone photoreceptor degeneration was found to be complete within the first postnatal weeks in *Rpe65*^−/−^ mice, whereas rod photoreceptor degeneration progressed slowly [8,9]. Early cone loss was reflected by a rapid decrease in cone-specific gene expression [10,11].

The gene coding for the interphotoreceptor matrix (IPM) proteoglycan 2 (*IMPG2*) was induced in retinas of *Rpe65*^−/−^ mice [11,12]. The IPM is a specialized extracellular matrix of fundamental importance to vision, e.g., in trafficking of retinoids and other metabolites between photoreceptors and the RPE, and in retinal adhesion or in photoreceptor outer segment recognition for phagocytosis [13]. It has been proposed that proteoglycans containing hyaluronic acid-binding motifs, e.g., IMPG2, IMPG1, and CD44, a cell surface adhesion molecule specifically localized in the Müller cell microvilli that oppose the IPM [14], stabilize a scaffold of hyaluronic acid in the IPM [15]. Interestingly, increased *CD44* mRNA levels have also been observed in *Rpe65*^−/−^ retinas [11].

Additionally, expression of the transmembrane neuronal proteoglycan with chondroitin sulfate (NGC: neuroglycan C; also called CALEB: chicken acidic leucine-rich EGF-like domain containing brain protein; CSPG5: chondroitin sulfate proteoglycan 5) has also been induced in *Rpe65*^−/−^ retinas [11,16,17]. Chondroitin sulfate side chains become attached to the NGC core protein in the developing rat cerebellum and retina, but not the adult ones [18,19]. NGC has therefore been described as a part-time proteoglycan. In the central nervous system, NGC has been found to be associated with both glial and neuronal surfaces [17]. In the retina, NGC is highly expressed on the axons of the nerve fiber layer and the inner plexiform layer at early postnatal stages (between P0 and P14), when active dendrite branching and conventional synapses between amacrine cells and ganglion cells can be observed in the inner retinal layers [19]. At late postnatal and adult stages (between P14 and P42), when synapse formation and dendrite branching are almost complete, NGC expression was found to be reduced [19]. NGC was localized to basal infoldings at P7 and to microvillis of the apical surface in the adult retina (P42) [19], indicating that high NGC expression in the RPE is differentially regulated during development. Neuronal depolarization of chick retinal cells in culture was shown to facilitate the processing of full-length NGC into a truncated transmembrane form and an ectodomain [20]. This activity-dependent ectodomain shedding exposed the EGF-like domain, located in the C-terminus of the ectodomain of NGC [20]. Interestingly, a recombinant ectodomain promoted neurite outgrowth from rat neocortical neurons in culture [21] and mediated dendritic tree and spine complexity in vivo [22].

The aim of this study was to validate and characterize the NGC expression during retinal degeneration in *Rpe65^-/-^* mice. Additionally, we assessed the expression of IMPG2 and CD44.

## Methods

### Animal handling

All experiments performed in this study were in accordance with the ARVO Statement for the Use of Animals in Ophthalmic and Vision Research and were approved by the Veterinary Service of the State of Valais (Switzerland). C57BL/6 mice (RCC, Basel, Switzerland) and *Rpe65*^−/−^ mice [7] were kept in a 12 h:12 h light-dark cycle with unlimited access to food and water until they were used for experiments.

### RNA preparation

Mice were killed by cervical dislocation. Eyes were enucleated, immobilized with 0.2 mm Austerlitz insect pins (Fine Science Tools, Heidelberg, Germany) on a Sylgard 184-filled cell culture dish (Dow Corning, Midland, MI), and covered with 1X PBS (phosphate-buffered saline: 154 mM NaCl, 1 mM KH_2_PO_4_ , 3 mM Na_2_HPO_4_ heptahydrate). Under a Leica 16MZF microscope (Leica Microsystems, Heerbrugg, Switzerland), the eyeball was sectioned below the ora serrata to remove cornea, lens, iris, and other attached tissues. The retina was then removed by cutting the optic nerve. For quantitative PCR experiments, pure RPE cells were obtained by trypsin-digestion from the posterior eyecup [23]. Briefly, the posterior eyecup comprised of RPE, choriocapillaris, and sclera was incubated in 0.2% trypsin (Invitrogen, Basel, Switzerland) for 1 h at 37 °C in a 5% CO2 atmosphere. Then, RPE cells were gently peeled off with forceps. For RT–PCR experiments, the sclera was dissociated from the RPE and attached choroidal tissue through homogenization with 18 gauge Sterican needles (Braun, Melsungen, Germany) in TRIzol (Invitrogen). Dissociated RPE with attached choroid was separated from the sclera by centrifugation at 1000x g and stored at −80 °C. Total RNA from the different dissected eye tissues was prepared according to manufacturer’s instructions, with prolonged centrifugation times to increase RNA recovery.

### Oligonucleotide microarray

The oligonucleotide microarray was previously described in detail [11]. Briefly, three retinas were pooled for each time point. Total RNA (1 μg) was used to generate doublestranded cDNA used as a template for biotinylated cRNA synthesis using Affymetrix GeneChip Expression 3′-Amplification Kit for IVT Labeling Kit (Affymetrix, Santa Clara, CA). Next, 20 μg of target cRNA were fragmented and hybridized on Affymetrix Mouse Genome 430 2.0 GeneChips, and washed chips were scanned on an Affymetrix GeneChip Scanner 3000 using the GCOS software (Affymetrix). Data normalization was performed using the Robust Multi-Array Analysis algorithm (RMA) as implemented in the GeneSpring 7.2 software (Agilent Technologies, Santa Clara, CA). Triplicates were performed for each condition studied. The intensity files corresponding to our raw data are deposited in NCBI Gene Expression Omnibus (GEO) database.

### PCR

Total RNA (2 μg) were used for reverse transcription (StrataScript™; Stratagene, La Jolla, CA). One-tenth of the reaction was used for subsequent PCR experiments. After an initial denaturation of 3 min at 94 °C, a 30 cycle PCR amplification was performed as follows: 30 s at 94 °C, 30 s at 60 °C, and 45 s at 72 °C.  Amplification was followed by a final elongation of 5 min at 72 °C (Taq PCR Master Mix ; Qiagen, Hilden, Germany). Expression of the ribosomal protein L8 (RL8) was used as internal standard [24]. PCR products were analyzed on 2% agarose gels. Quantitative PCR was performed on an Mx3000p sequence analyzer using Brilliant®SYBR®Green qPCR Master Mix I (Stratagene). Experimental data points with cycle threshold (C_t_) values above 30 were not considered for data analysis. Primers are listed in Table 1.

**Table 1:** Primers for PCR.

**Gene/ isoform**	**Forward primer (5′-3′)**	**Reverse primer (5′-3′)**	**GenBank accession number**	**Location (nt)**
*NGC*	GACTGAGAATACCAAGCTGC	TTGGGTGACATGGAGTTCTG	NM_013884	1500/1700
*NGC-I*	AGTGCTGCTGCTTCTGGGGGTCA	TTATCATGGACAGCAGGGGA	NM_013884	192/644
*NGC-II*	ATTTGGGGCGGGAAACCATA	TTATCATGGACAGCAGGGGA	NM_013884	−98/644
*NGC-III*	CTCCACAACGACAACTTCTC	AGAGGGTCCTGGATTTTGTG	NM_013884	1552/1643
*IMPG2*	CTTCTGCTGTCGTCTTCTTC	CCAATCACCTCTTCACTAGC	BC048863	3544/3769
*CD44*	CTCAGATTCCAGAATGGCTC	TCAGCTGTCATACACTGGTC	NM_009851	2367/2616
*RL8*	ACTGGACAGTTCGTGTACTG	GCTTCACTCGAGTCTTCTTG	NM_012053	271/469

The location of the PCR amplification products is indicated with respect to nucleotide numbering of the indicated GenBank accession numbers. The location of the *NGC-II*-specific forward primer was according to the publicly available genomic sequence of chromosome 9 (ensembl, release 49) and by defining the A of the initial ATG of the coding sequence as nt 1.

### In situ hybridization

Eyes were enucleated, rinsed in diethyl-pyrocarbonate (DEPC)-treated 1X PBS and fixed for 2 h with 4% paraformaldehyde-1X PBS-DEPC at 4 °C. After cryoprotection by immersion in 30% sucrose-1X PBS-DEPC, overnight at 4 °C, eyes were embedded in freezing compound (30% albumin/3% gelatine in DEPC-treated 1X PBS). Eyes were sectioned at −21 °C on a Leica CM1900 cryostat, and 12 μm sections were recovered on SuperFrost®Plus microscope slides (Menzel Gläser, Braunschweig, Germany) pretreated with Vectabond (Vector Laboratories, Burlingame, CA). PCR fragments of *NGC* and *IMPG2* cDNAs, amplified with primers shown in Table 1, were subcloned into the pGEM®-T Easy Vector (Promega, Madison, WI). Plasmids were linearized with XbaI or NcoI for probe synthesis. Digoxygenin (DIG)-labeled sense and antisense probes were tested by immunodot blotting and equal amounts of probe used in the experiment [25]. In situ hybridizations were performed as previously described, including a carbamoylation step in active 0.1% DEPC-1X PBS at a hybridization temperature of 48 °C [25].

### Immunohistochemistry

Eyes were enucleated and fixed in 4% paraformaldehyde-1X PBS for 2 h at 4 °C. After cryoprotection by immersion in 30% sucrose-1X PBS overnight at 4 °C, eyes were embedded in freezing compound (30% albumin/3% gelatine in 1X PBS). For immunohistochemistry, 12 μm cryosections were collected on Superfrost®Plus glass slides (Menzel,). Sections were dried at room temperature for at least 1 h, quickly hydrated with 1X PBS, and blocked for 1 h in 1X PBS containing 2% goat serum and 0.2% Triton X-100. A rabbit polyclonal serum raised against the extracellular domain of rat NGC was diluted at 1:500 in blocking solution and incubated overnight at 4 °C [26]. Sections were then briefly rinsed twice with blocking solution and washed once for 5 min. Secondary antibodies conjugated to Alexa Fluor 594 (Molecular Probes, Invitrogen) were diluted at 1:1,000 and incubated for 1 h at room temperature in the dark. Sections were rinsed briefly twice in 1X PBS then washed three times for 5 min. Parts of sections were stained with DAPI to visualize nuclei. To stain cone photoreceptor outer segments, we used 20 μg/ml fluorescein-conjugated peanut agglutinin (Sigma-Fluka, Buchs, Switzerland). Slides were washed three times for 5 min in 1X PBS, before mounting in Cityfluor (Cityfluor Ltd., London,UK).

### Western blotting

On ice, four mouse retinas were homogenized with a plastic pestle in 200 μl of a buffer containing 100 mM NaCl, 50 mM Tris pH 7.5, 1 mM EDTA, 0.1% Triton X-100, 10 mM NaF, and freshly added proteinase inhibitors (Complete; Roche, Basel, Switzerland). Protein extracts were stored at −80 °C. For western blotting, 20 μg of protein extracts were separated on a 5% stacking gel and a 6% resolving gel. Proteins were transferred onto a polyvinlyidenfluoride (PVDF) membrane (Westran® Clear Signal; GE Healthcare, Piscataway, NJ). Membranes were blocked for 1 h in TBS-Tween containing 5% nonfat dry milk. The rabbit polyclonal anti-NGC antibody [26] and the rabbit polyclonal anti-α-tubulin (H-300) antibody (Santa Cruz Biotechnology, Santa Cruz, CA) were diluted 1:5,000. The secondary ECL™ donkey anti-rabbit IgG horseradish peroxidase-conjugated antibody was diluted 1:25,000 (GE Healthcare). Proteins were detected by chemiluminescence (ECL Plus Western Blotting Detection System, GE Healthcare) on a Hyperfilm™ ECL (GE Healthcare). Signals were quantified with Gelquant software Version 2.7.0 (DNR Imaging Systems Ltd., Jerusalem, Israel).

### Statistical analysis

Statistical analysis was performed on normalized cycle threshold values for quantitative PCR and on raw densitometric data for western blot analysis. Data were analyzed by a two-way ANOVA, using genotype and age factors (Graphpad Prism 4.0.2; GraphPad Software Inc., La Jolla, CA).

## Results

### Upregulation of transcripts coding for IPM proteins in *Rpe65*^−/−^ retinas

**Figure 1:**
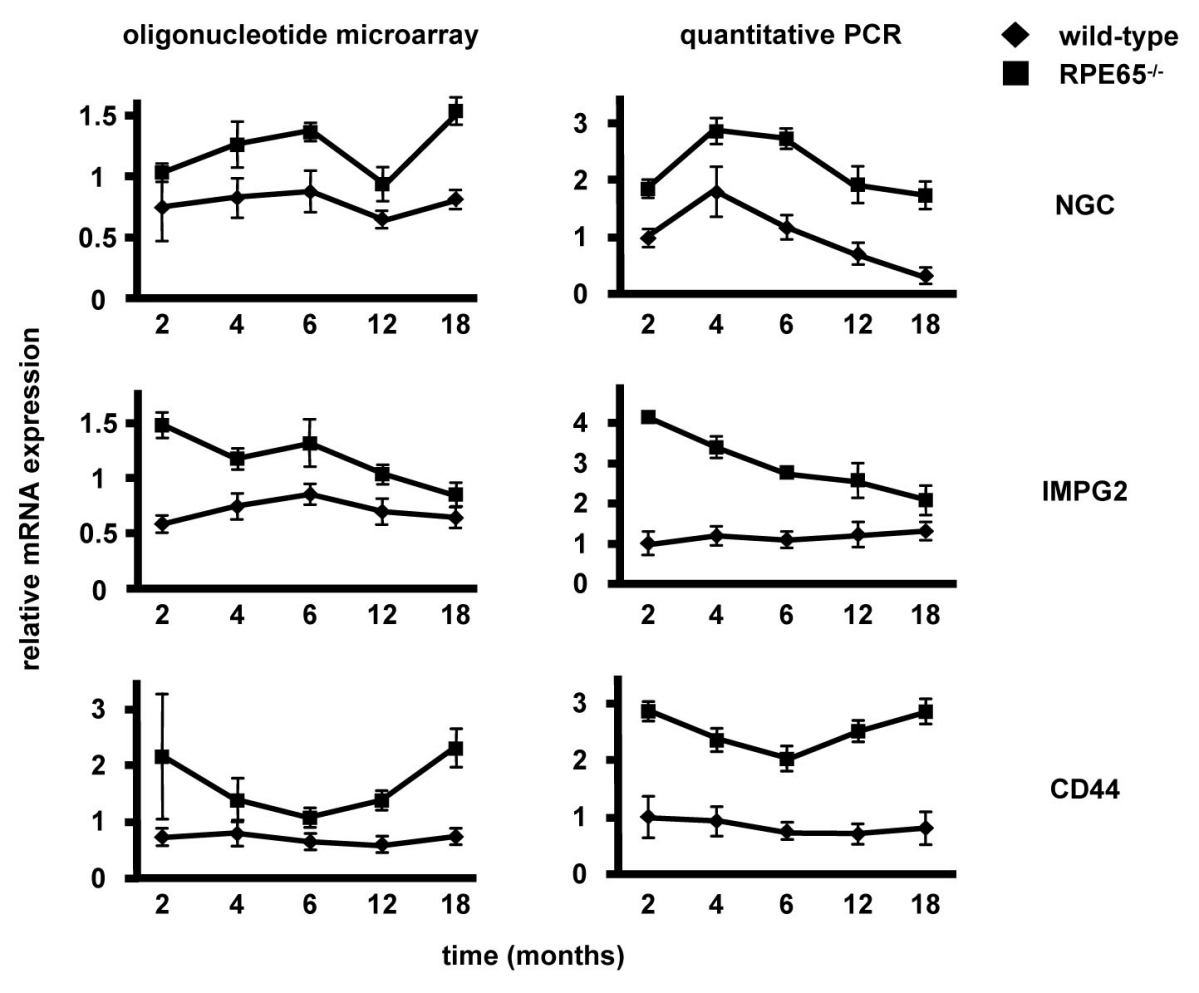
Retinal expression of *NGC*, *IMPG2*, and *CD44* mRNAs in *Rpe65*^−/−^ mice. mRNA expression from wild-type (diamonds) and *Rpe65*^−/−^ retinas (squares) was assessed at 2, 4, 6, 12, and 18 months by oligonucleotide microarray (left panels, n=3) and validated by quantitative PCR (right panels, n=6). A complete description of the oligonucleotide microarray data at 2, 4, and 6 months has been published in [11]. For quantitative PCR, mRNA expression was normalized to *RL8* mRNA expression. Samples from 2-month-old wild-type mice were arbitrarily set to 1. Fold inductions +/− SEM are shown. It was found by two-way ANOVA that mRNA expression levels were significantly different between genotypes for all time points (p<0.01).

In an initial gene expression profiling experiment using Affymetrix® oligonucleotide microarrays, 534 genes were differentially expressed in *Rpe65*^−/−^ mouse retinas after two, four, and six months of disease progression [11]. We extended the time-course to up to 18 months (Figure 1, left panels). The genes coding for the reported IPM proteins IMPG2 and CD44 were induced by over 2 fold during disease progression. Additionally, we observed an up to 1.7 fold induction of *NGC* mRNA expression in *Rpe65*^−/−^ retinas. We validated the oligonucleotide microarray data by quantitative PCR (Figure 1, right panels). At all time points, *NGC* mRNA expression was induced by over 2 fold in *Rpe65*^−/−^ retinas versus wild-type levels (p<0.01). Additionally, there was a significant decrease in *NGC* mRNA expression over time in both wild-type and *Rpe65*^−/−^ retinas (p<0.01), consistent with previous observations in developing and adult retinas [19]. *IMPG2* mRNA levels were induced in *Rpe65*^−/−^ retinas by 4.2 fold at two months, and decreased progressively to less than 2 fold at 18 months. The mRNA levels for *CD44* were induced in *Rpe65*^−/−^ retinas by 2.9 fold at two months and remained elevated during disease progression up to 18 months.

### Differential expression of *NGC* mRNA isoforms in the eye

**Figure 2:**
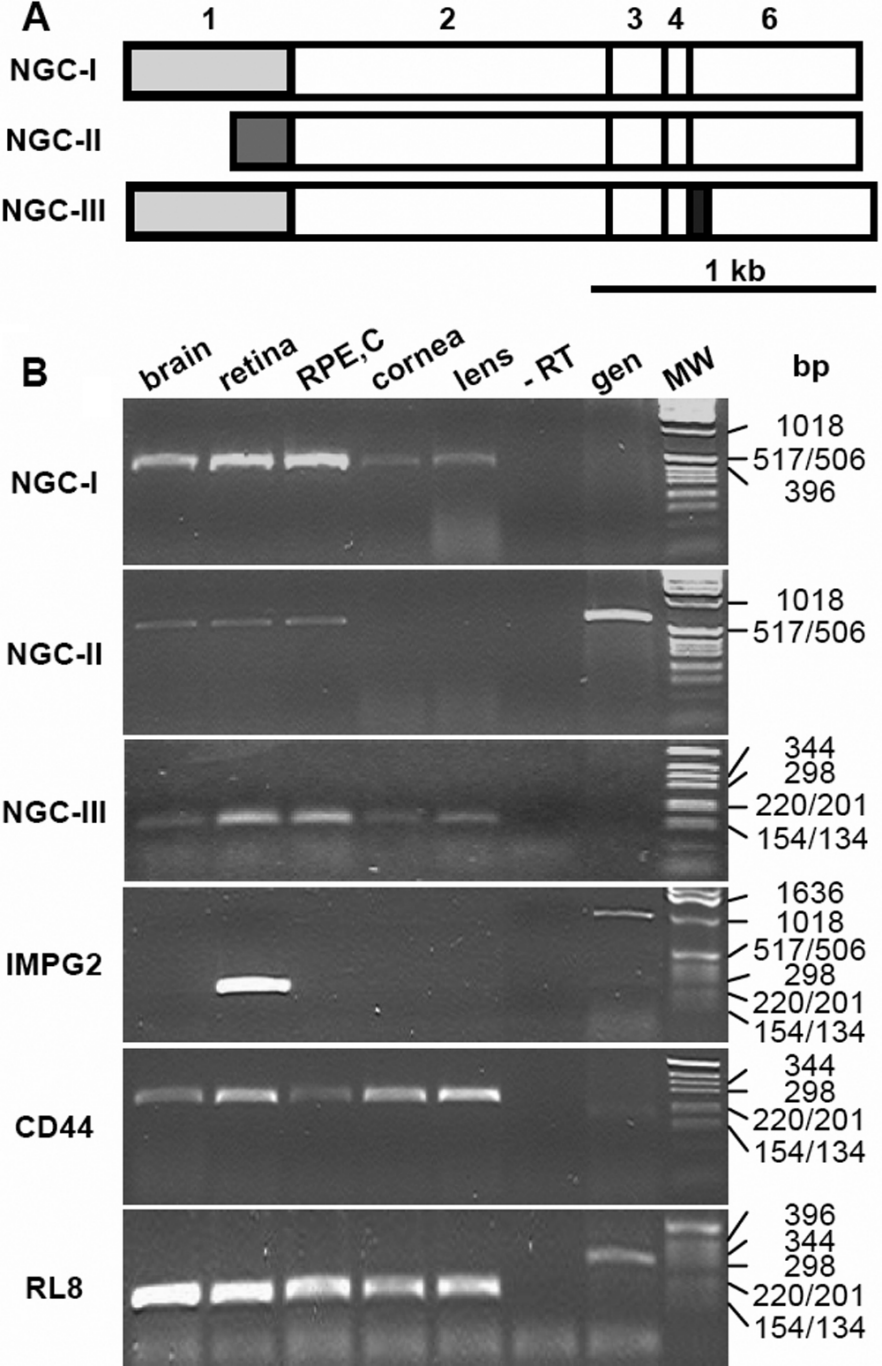
Differential expression of *NGC* mRNA isoforms in the adult mouse eye. **A:** Schematic representation of mouse *NGC* isoforms. Exons are numbered above the scheme. The *NGC-I* isoform contains exon 1 (light gray), that is replaced through alternative splicing by exon 1’ (dark gray) in the *NGC-II* isoform. An additional exon 5 (black) is present in isoform *NGC-III*. **B:** *NGC* isoform mRNA expression in adult mouse tissues. RT–PCR experiments were performed on pooled tissues from four different eyes of 8-week-old mice. One representative experiment out of two is shown. *RL8* mRNA expression was used as an internal standard. Abbreviations: retinal pigment epithelium with attached choroid (RPE,C); no template (-RT); 200 ng mouse genomic DNA (gen); DNA molecular weight marker X (MW). Only relevant band sizes are indicated. Specific cDNA amplification yielded products of 452 bp for *NGC-I*, 595 bp for *NGC-II*, 178 bp for NGC-III, 226 bp for *IMPG2*, 250 bp for *CD44*, and 199 bp for *RL8*. Amplification on genomic DNA yielded products of 595 bp for *NGC-II*, 1192 bp for *IMPG2*, and 336 bp for *RL8*.

To date, three different *NGC* isoforms have been reported in mice [18]. They were generated through alternative exon usage in 5′. Exon 1 present in *NGC-I* can be replaced by an intronic sequence (exon 1’) and yield isoform *NGC-II* (Figure 2A). Additionally, the presence of the alternatively spliced exon 5 results in isoform *NGC-III*. All three *NGC* isoforms were detected in adult mouse brain and eye by isoform-specific PCR (Figure 2B). *NGC-I* levels were highest in the RPE, the retina, and the brain; they were lower in the lens and the cornea. For *NGC-II*, the size of the PCR amplification products from mouse genomic DNA and cDNA were identical. *NGC-II* was barely detected in brain, retina and RPE, but a strong signal was present with mouse genomic DNA. *NGC-III* was detected in a similar pattern than *NGC-I*. *IMPG2* was selectively expressed in the retina, whereas *CD44* was detected in all analyzed tissues. Genomic fragments were also amplified on mouse genomic DNA with primers for *RL8* and *IMPG2*. In contrast to what had been observed for *NGC-II*, these signals were weaker than those amplified from cDNA templates, thus suggesting low* NGC-II* mRNA expression levels. Furthermore, contamination of the cDNA templates by genomic DNA could be excluded by the absence of amplification products corresponding to the *RL8* genomic fragments.

### Differential expression of *NGC* mRNA isoforms in *Rpe65*^−/−^ mice

**Figure 3:**
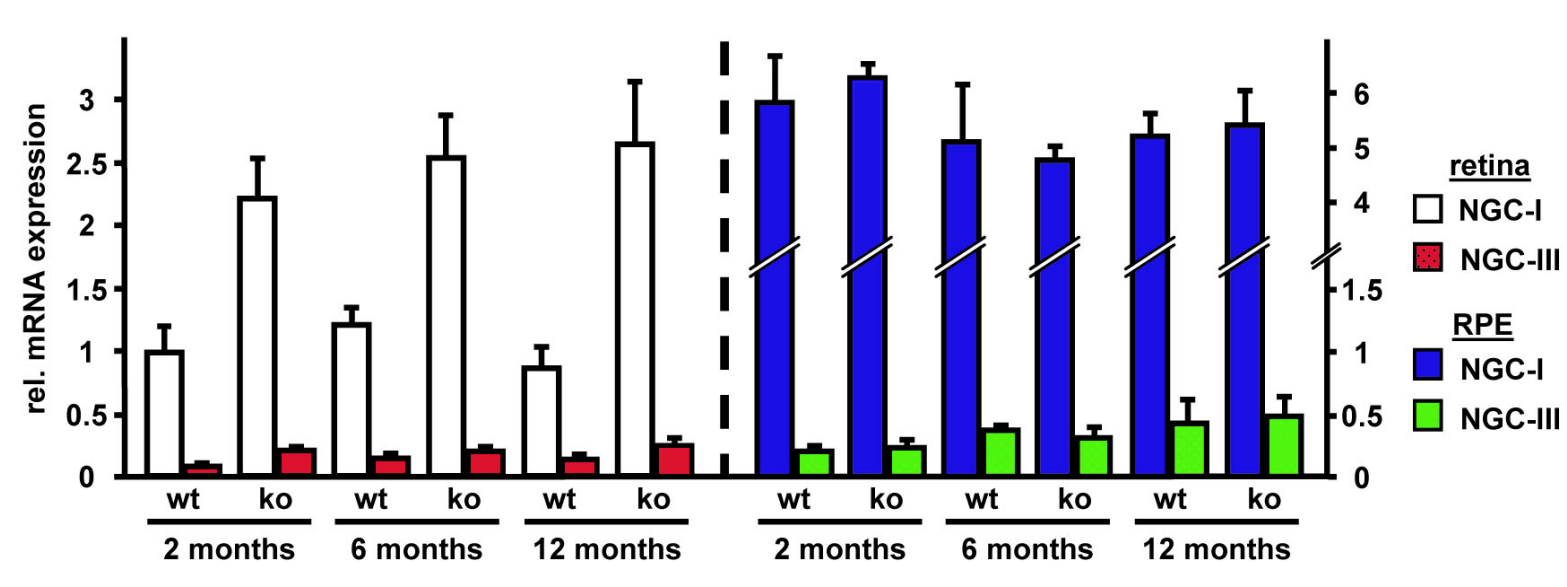
Differential expression of *NGC* mRNA isoforms in *Rpe65^−/−^* mice. *NGC-I* (left bars) and *NGC-III* (right bars) mRNA expression was assessed by quantitative PCR on total RNA extracted from wild-type (wt) and *Rpe65^−/−^* mice (ko) at 2, 6, and 12 months (n=3). Left panel shows retinal samples and right panel RPE cell samples. Retinal *NGC-I* mRNA expression (white bars) was compared to *NGC-III* mRNA levels (red bars). Similarly, RPE *NGC-I* mRNA expression (blue bars) was compared to *NGC-III* mRNA levels (green bars). Retinal *NGC-I* mRNA expression of 2-month-old wild-type mice was arbitrarily set to 1. For both panels, fold inductions±SEM are shown. By two-way ANOVA retinal *NGC-I* and *NGC-III* mRNA levels were significantly increased in *Rpe65^−/−^* mice (p<0.01). Increase in *NGC-III* mRNA expression in the RPE was also significant at 6 and 12 months (p<0.05).

To assess isoform-specific *NGC* mRNA expression, we performed quantitative PCR experiments on retinas and RPE cells of 2-, 6- and 12-month-old wild-type and *Rpe65*^−/−^ mice (Figure 3). *NGC-I* expression was over 2 fold induced in *Rpe65*^−/−^ retinas at all time points. NGC-I transcripts were around 5 fold to 6 fold more abundant in the RPE than in the retina, but expression levels were not increased in *Rpe65*^−/−^ mice. *NGC-II* levels were comparable between wild-type and *Rpe65*^−/−^ mice, but at least 16.9 old lower than those of *NGC-I*, with cycle threshold values beyond our threshold of confidence (Ct>34; see Methods; data not shown). In wild-type retinas, *NGC-III* mRNA levels were about 10 fold lower than *NGC-I* levels. They were increased in *Rpe65*^−/−^ retinas, to a similar extent than that observed for *NGC-I* (p<0.01). *NGC-III* mRNA levels were low in the RPE, but became significantly increased over age (p<0.05).

### *NGC* mRNA expression in adult wild-type and *Rpe65*^−/−^ retinas

**Figure 4:**
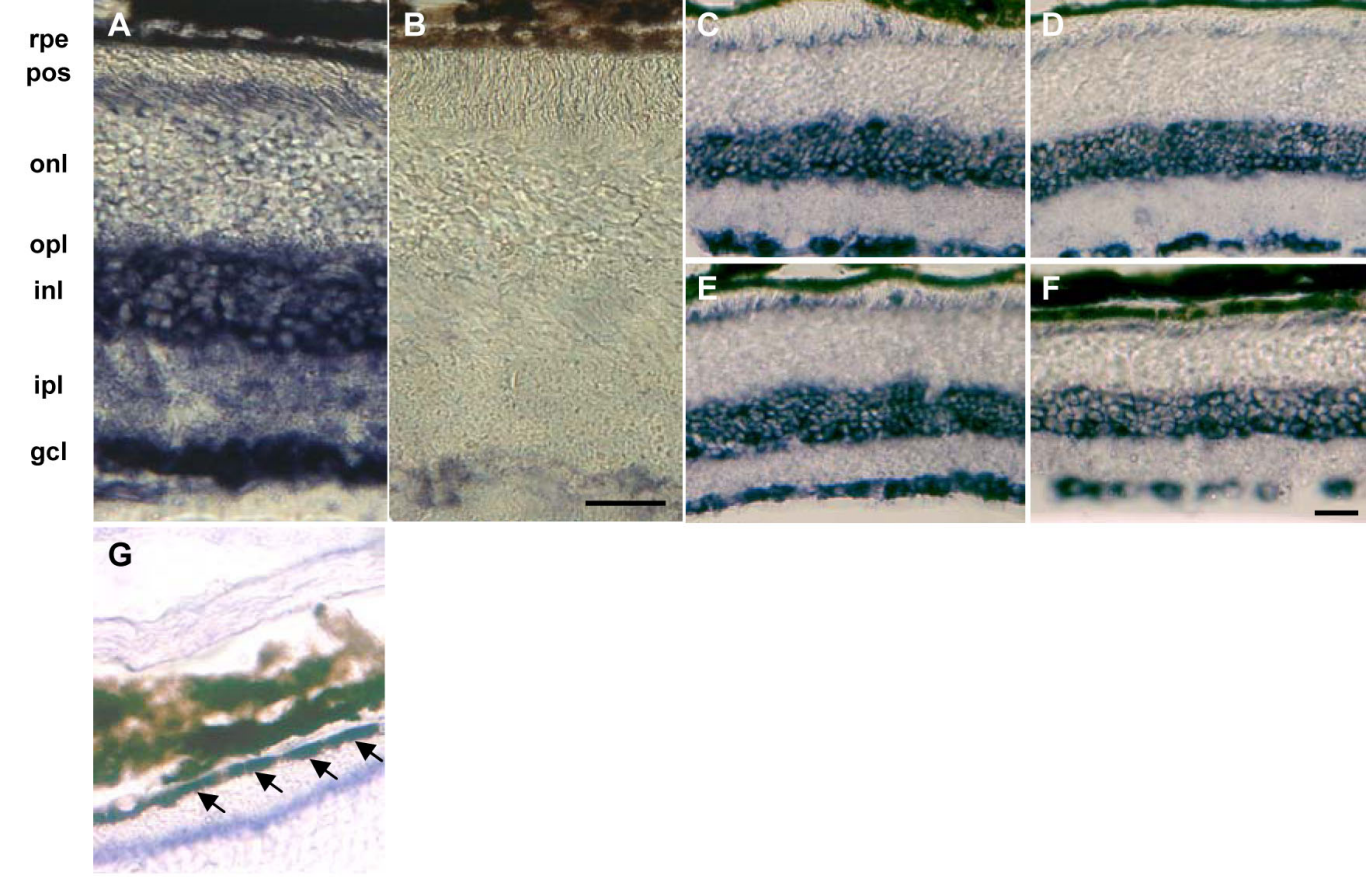
In situ hybridization of *NGC* transcripts. In situ hybridization studies were performed on retinal sections of 2-month-old wild-type mice with antisense (**A**) and control sense (**B**) probes. Retinal sections of wild-type (**C,D**) and *Rpe65*^−/−^ (**E,F**) mice at 2 (**C,E**) and 6 months of age (**D,F**) were hybridized with the antisense probe to detect NGC transcripts. NGC mRNA is expressed in the RPE cell layer (**G**; arrows). Abbreviations: retinal pigment epithelium (rpe): photoreceptor outer segments (pos); outer nuclear layer (onl); outer plexiform layer (opl); inner nuclear layer (inl); inner plexiform layer (ipl); retinal ganglion cell layer (gcl). Scale bars represents 30 μm (**A,B**) and 40 μm (**C-F**).

We analyzed *NGC* mRNA expression by in situ hybridization (Figure 4) to exclude the possibility that the observed increase in *NGC* mRNA expression in *Rpe65*^−/−^ retinas was due to de novo expression in retinal cell populations that do not express *NGC* in wild-type mice. *NGC* mRNA was highly expressed in the ganglion cell layer, in the inner nuclear layer, around the outer limiting membrane, and in the RPE (Figure 4A,G). It was also detected at low levels in the inner plexiform layer and the outer nuclear layer (Figure 4A). We did not observe ectopic *NGC* mRNA expression during disease progression, not even in 6-month-old *Rpe65*^−/−^ mice, where the outer retina was disorganized [7] (Figure 4C-F).

**Figure 5:**
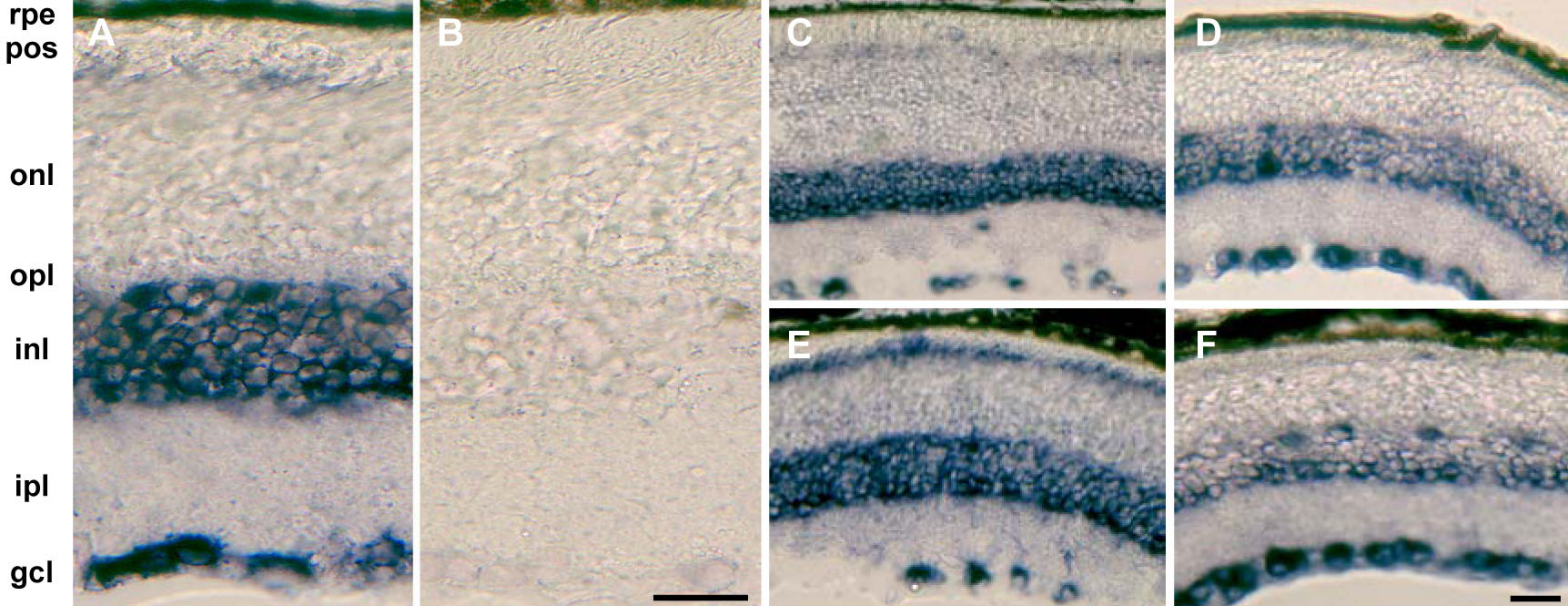
In situ hybridization of *IMPG2* transcripts. In situ hybridization studies were performed on retinal sections from 2-month-old wild-type mice with antisense (**A**) and control sense (**B**) probes. Retinal sections of wild-type (**C,D**) and *Rpe65*^−/−^ (**E,F**) mice at 2 (**C,E**) and 6 months of age (**D,F**) were hybridized with the antisense probe to detect *IMPG2* transcripts. Abbreviations: retinal pigment epithelium (rpe); photoreceptor outer segments (pos);  outer nuclear layer (onl): outer plexiform layer (opl); inner nuclear layer (inl); inner plexiform layer (ipl); retinal ganglion cell layer. Scale bars equal 30 μm (**A,B**) and 40 μm (**C-F**).

We also examined mRNA expression of *IMPG2* during disease progression (Figure 5). In wild-type retinas, the mRNA expression pattern of *IMPG2* was comparable to that of *NGC* (Figure 5A). In 2-month-old *Rpe65*^−/−^ retinas, increased *IMPG2* mRNA expression was observed at the outer limiting membrane (Figure 5E). At six months, *IMPG2* mRNA expression was decreased at the outer limiting membrane and the inner nuclear layer in *Rpe65*^−/−^ retinas (Figure 5F), and, to some extent, in wild-type retinas (Figure 5D).

### Upregulation of NGC protein expression in *Rpe65*^−/−^ retinas

**Figure 6:**
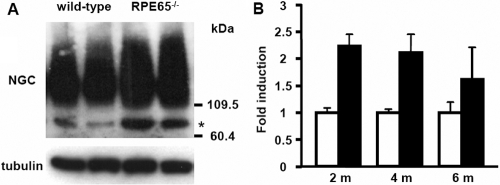
NGC protein expression during retinal degeneration in *Rpe65*^−/−^ mice. **A:** In the present study, 20 μg of total protein extracts from four pooled retinas of 2-month-old wild-type and *Rpe65*^−/−^ mice were resolved on a 6% SDS–PAGE and analyzed by western blot. Posttranslational modifications of the NGC full-length protein resulted in a signal for NGC under appearance of a smear. The asterisk marks the shedded NGC ectodomain of about 75 kDa. **B:** Total protein extracts were prepared from one retina of 2-, 4-, and 6-months (m)-old wild-type (white bars) and *Rpe65^-/-^* mice (black bars). NGC expression was assessed by western blot and subsequently quantified (n=3). The sum of NGC full-length and ectodomain signal intensities were normalized to α-tubulin expression. NGC expression was statistically different between wild-type and *Rpe65^-/-^* retinas at 2 and 4 months, but not 6 months of age, as assessed by two-way ANOVA (p<0.01) and by Student’s */t/*-test.

To test whether the observed increase in *NGC* mRNA expression correlated with an increase in NGC protein levels, we performed western blot analysis on total retinal protein extracts of 2-, 4- and 6-month-old wild-type and *Rpe65*^−/−^ mice (Figure 6). An antisera raised against the extracellular part of human NGC detected a protein as a smear from about 120 kDa upwards, corresponding to the NGC core protein of 120 kDa with multiple posttranslational modifications [16,26,27] (Figure 6A). In retinal protein extracts of 2-month-old mice, and to a lesser extent in 4-month-old mice, a marked increase in the activity-cleaved 75 kDa-ectodomain of NGC was observed (Figure 6A; data not shown). NGC protein levels, i.e., the sum of full-length NGC and the ectodomain, were over 2 fold induced in retinas of *Rpe65*^−/−^ mice at 2 and 4 months of age, but no significant difference was detected at 6 months (Figure 6B).

### NGC is highly expressed in the outer retina

**Figure 7:**
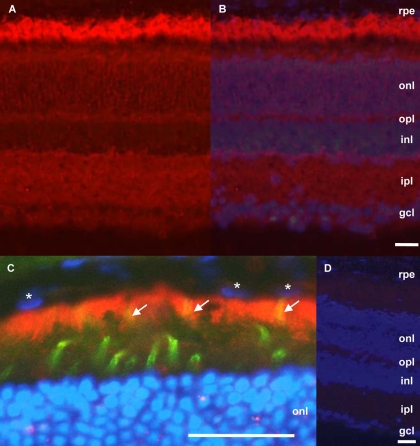
NGC protein expression in the adult mouse retina. Immunohistochemical analysis was performed with an antiserum raised against the extracellular part of NGC (red) on retinal sections of 2-month-old wild-type mice (**A**). Nuclei were stained in blue with DAPI, and images were merged (**B**). As a negative control, serum from a nonimmunized rabbit was used; the nuclei were stained with DAPI, and the images were merged (**D**). **C:** NGC is predominantly expressed at the apical side of RPE cells. NGC appears in red, nuclei are in blue, and outer segments of cone photoreceptor cells are in green. Note cone outer segments surrounded by microvilli of RPE cells in yellow (arrows). This image was obtained by filtering the intensity of the fluorescence down to 6.25% for the red channel, by fixing the one of the green channel at 100%. Stars denote three nuclei of RPE cells. Abbreviations: retinal pigment epithelium (rpe); outer nuclear layer (onl); outer plexiform layer (opl); inner nuclear layer (inl); inner plexiform layer (ipl); ganglion cell layer (gcl). Scale bars equal 30 μm.

The retinal NGC expression pattern detected by immunohistochemistry with the antisera raised against the entire extracellular domain of NGC [26] was comparable to that detected with an antisera against a partial ectodomain [19,28]. NGC was enriched in all neurite-containing retinal layers of adult mouse retina (Figure 7A,B). NGC was expressed in the nerve fiber layer, the inner plexiform layer, and the outer plexiform layer, but was absent in the cell bodies of ganglion cells and in the inner nuclear layer. In the outer neural retina, NGC staining was intense around the outer limiting membrane and was associated with longitudinal structures in the outer nuclear layer (Figure 7A,B). Consistent with the mRNA expression, the highest levels of NGC expression were detected in the RPE (Figure 7C). Counterstaining with a cone-specific lectin showed that NGC immunoreactivity was highest at the apical membranes of RPE cells, surrounding the outer segments of the photoreceptors (Figure 7C).

## Discussion

In the present work, we showed that *NGC*, *IMPG2*, and *CD44* mRNA expression was induced during retinal degeneration in *Rpe65*^−/−^ mice. The amplitude of fold-inductions were similar to those observed for proteoglycans and CD44 in other mouse models of retinal degeneration, namely rd1 (rodless retina), rds (retinal degeneration slow, rd2), and rhodopsin knockout (*Rho*^−/−^) mice [29-31]. Upregulation of extracellular matrix proteins expressed in the IPM might therefore be a general mechanism observed in retinal degeneration.

The induction of *CD44* gene expression had been proposed to be an attempt of the Müller cells to strengthen the IPM, thereby counteracting the degenerative process [29]. Elevated *IMPG2* mRNA levels early in retinal degeneration might also be circumstantial evidence of the importance of chondroitin sulfate proteoglycans in the IPM to maintain functional photoreceptors. Indeed, both the inhibition of chondroitin sulfate proteoglycan synthesis by intravitreal injections of p-nitrophenyl-β-D-xylopyranoside and the impairment of β-glucuronidase-mediated lysosomal degradation of chondroitin sulphate, resulted in photoreceptor degeneration [32,33]. Notably, at 18 months, when the photoreceptors have almost completely disappeared in the *Rpe65*^−/−^ mice, *IMPG2* mRNA levels were down to levels observed in age-matched wild-type retinas. Consistently, the decrease in *IMPG2* mRNA expression appeared to start from the outer retina toward the inner retina, as suggested by in situ hybridization on 6-month-old retinas.

In the present work, we identified NGC as an additional chondroitin sulfate proteoglycan expressed in the IPM during retinal degeneration in *Rpe65*^−/−^ mice. *NGC-I* was the most highly expressed isoform in the retina and in the RPE. Additionally, mainly *NGC-I* mRNA expression was induced during retinal degeneration in *Rpe65*^−/−^ mice. The low *NGC-II* mRNA expression in brain, retina, and RPE could not be assessed by quantitative PCR. It has to be mentioned at this point, that the presence of a NGC-II protein in the central nervous system has not been assessed to date [26]. The isoform *NGC-III* was present at low levels both in the retina and the RPE. Taken together, our results define NGC-I as the major isoform in the retina and the RPE.

At the protein level, we observed a significant induction of NGC expression in retinas as old as four months. Most interestingly, we observed an increase in the shedded ectodomain of NGC in 2- and 4-month-old retinas. This EGF-like-domain-containing ectodomain is sufficient to promote neurite outgrowth from rat neocortical neurons in culture [21]. Additionally, the full-length NGC is a critical modulator of dendritic branching and spine formation both in cultured primary neurons and in the mouse cortex [17,22]. The induction of NGC expression in neurite-containing retinal layers could therefore be an attempt to maintain proper synaptic transmission in the degenerating retina. Recently, tissue inhibitor of metalloproteinases 2 (TIMP-2) and TIMP-3 have been identified in vitro as inhibitors of NGC ectodomain shedding [34]. It will be interesting to assess the in vivo activity of TIMP-2 and -3 in *RPE65^-/-^* retinas and test any physiological effect on ectodomain shedding.

In the adult eye, NGC was most abundantly expressed at the apical membrane of the RPE. However, its transcript levels were not differentially regulated during retinal degeneration, suggesting the presence of tissue-specific regulatory elements in the *NGC* promoter. Constitutive expression of NGC at the apical membrane of RPE cells might be necessary to modulate the physiologic interactions between the RPE and the photoreceptors, e.g. the phagocytosis of photoreceptor outer segments in the adult retina [19]. The availability of *NGC*^−/−^ mice will allow a detailed analysis of the importance of NGC in maintaining the retinal neuronal network during retinal degeneration in *Rpe65^−/−^* mice [20].
